# Nicotine receptor partial agonists for smoking cessation

**DOI:** 10.1590/S1516-31802012000500014

**Published:** 2012-11-13

**Authors:** Kate Cahill, Lindsay F. Stead, Tim Lancaster

## Abstract

**BACKGROUND:**

Nicotine receptor partial agonists may help people to stop smoking by a combination of maintaining moderate levels of dopamine to counteract withdrawal symptoms (acting as an agonist) and reducing smoking satisfaction (acting as an antagonist).

**OBJECTIVES:**

The primary objective of this review is to assess the efficacy and tolerability of nicotine receptor partial agonists, including cytisine, dianicline and varenicline for smoking cessation.

**SEARCH METHODS:**

We searched the Cochrane Tobacco Addiction Group’s specialised register for trials, using the terms (‘cytisine’ or ‘Tabex’ or ‘dianicline’ or ’varenicline’ or ’nicotine receptor partial agonist’) in the title or abstract, or as keywords. The register is compiled from searches of MEDLINE, EMBASE, PsycINFO and Web of Science using MeSH terms and free text to identify controlled trials of interventions for smoking cessation and prevention. We contacted authors of trial reports for additional information where necessary. The latest update of the specialized register was in December 2011. We also searched online clinical trials registers.

**SELECTION CRITERIA:**

We included randomized controlled trials which compared the treatment drug with placebo. We also included comparisons with bupropion and nicotine patches where available. We excluded trials which did not report a minimum follow-up period of six months from start of treatment.

**DATA COLLECTION AND ANALYSIS:**

We extracted data on the type of participants, the dose and duration of treatment, the outcome measures, the randomization procedure, concealment of allocation, and completeness of follow-up. The main outcome measured was abstinence from smoking at longest follow-up. We used the most rigorous definition of abstinence, and preferred biochemically validated rates where they were reported. Where appropriate we pooled risk ratios (RRs), using the Mantel-Haenszel fixed-effect model.

**MAIN RESULTS:**

Two recent cytisine trials (937 people) found that more participants taking cytisine stopped smoking compared with placebo at longest follow-up, with a pooled RR of 3.98 (95% confidence interval (CI) 2.01 to 7.87). One trial of dianicline (602 people) failed to find evidence that it was effective (RR 1.20, 95% CI 0.82 to 1.75). Fifteen trials compared varenicline with placebo for smoking cessation; three of these also included a bupropion treatment arm. We also found one open-label trial comparing varenicline plus counselling with counselling alone. We found one relapse prevention trial, comparing varenicline with placebo, and two open-label trials comparing varenicline with nicotine replacement therapy (NRT). We also included one trial in which all the participants were given varenicline, but received behavioural support either online or by phone calls, or by both methods. This trial is not included in the analyses, but contributes to the data on safety and tolerability. The included studies covered 12,223 participants, 8100 of whom used varenicline. The pooled RR for continuous or sustained abstinence at six months or longer for varenicline at standard dosage versus placebo was 2.27 (95% CI 2.02 to 2.55; 14 trials, 6166 people, excluding one trial evaluating long term safety). Varenicline at lower or variable doses was also shown to be effective, with an RR of 2.09 (95% CI 1.56 to 2.78; 4 trials, 1272 people). The pooled RR for varenicline versus bupropion at one year was 1.52 (95% CI 1.22 to 1.88; 3 trials, 1622 people). The RR for varenicline versus NRT for point prevalence abstinence at 24 weeks was 1.13 (95% CI 0.94 to 1.35; 2 trials, 778 people). The two trials which tested the use of varenicline beyond the 12-week standard regimen found the drug to be well-tolerated during long-term use. The main adverse effect of varenicline was nausea, which was mostly at mild to moderate levels and usually subsided over time. A meta-analysis of reported serious adverse events occurring during or after active treatment and not necessarily considered attributable to treatment suggests there may be a one third increase in the chance of severe adverse effects among people using varenicline (RR 1.36; 95% CI 1.04 to 1.79; 17 trials, 7725 people), but this finding needs to be tested further. Post-marketing safety data have raised questions about a possible association between varenicline and depressed mood, agitation, and suicidal behaviour or ideation. The labelling of varenicline was amended in 2008, and the manufacturers produced a Medication Guide. Thus far, surveillance reports and secondary analyses of trial data are inconclusive, but the possibility of a link between varenicline and serious psychiatric or cardiovascular events cannot be ruled out.

**AUTHORS’ CONCLUSIONS:**

Cytisine increases the chances of quitting, although absolute quit rates were modest in two recent trials. Varenicline at standard dose increased the chances of successful long-term smoking cessation between two- and threefold compared with pharmacologically unassisted quit attempts. Lower dose regimens also conferred benefits for cessation, while reducing the incidence of adverse events. More participants quit successfully with varenicline than with bupropion. Two open-label trials of varenicline versus NRT suggested a modest benefit of varenicline but confidence intervals did not rule out equivalence. Limited evidence suggests that varenicline may have a role to play in relapse prevention. The main adverse effect of varenicline is nausea, but mostly at mild to moderate levels and tending to subside over time. Possible links with serious adverse events, including serious psychiatric or cardiovascular events, cannot be ruled out. Future trials of cytisine may test extended regimens and more intensive behavioural support. There is a need for further trials of the efficacy of varenicline treatment extended beyond 12 weeks.

**This is the abstract of a Cochrane Review published in the Cochrane Database of Systematic Reviews (CDSR) 2012, issue 4, DOI:** 10.1002/14651858.CD006103.pub6 (http://onlinelibrary.wiley.com/doi/10.1002/14651858.CD006103.pub6/abstract). For full citation and authors’ details see reference 1

**The full text is freely available from:** http://www.cochranejournalclub.com/smoking-cessation-clinical/pdf/CD006103.pdf
